# Application of real-time B-mode ultrasound in posterior decompression and reduction for thoracolumbar burst fracture

**DOI:** 10.3892/etm.2013.1257

**Published:** 2013-08-07

**Authors:** WU-PENG YANG, ZHE WANG, NAI-QI FENG, CHUN-MEI WANG, SHAO-LONG DU

**Affiliations:** Department of Orthopaedics, The Ordos Center Hospital, The Ordos Clinic Medical College, Inner Mongolia Medical University, Ordos, Inner Mongolia Autonomous Region 017000, P.R. China

**Keywords:** real-time B-mode ultrasound, thoracolumbar burst fracture, posterior decompression, reduction

## Abstract

This study aimed to investigate the role of real-time B-mode ultrasound in posterior decompression and reduction and to observe the signal changes in spinal cord blood flow in a thoracolumbar burst fracture (TBF). Between February 2004 and December 2008, 138 patients with TBF were divided into group A (108 cases) and group B (30 cases). In group A, under the assistance of real-time B-mode ultrasound, posterior decompression and fracture piece reduction were performed, and we observed the signal changes in spinal cord blood flow. In group B, posterior fenestration was combined with pushing the fracture piece into the fractured vertebral body using an L-shaped operative tool. Presurgical and postsurgical recovery of neurological function was evaluated according to American Spinal Injury Association (ASIA) standards, and the range of spinal decompression was determined by measuring the proportion of encroached fracture piece in the spinal canal (spinal stenosis rate) on the computed tomography (CT) image. In group A, 12 patients had a grade A spinal injury according to the Frankel grading system, and there were six cases without neurological recovery. In the other patients, neurological function increased by 1–3 grades. There were no aggravated spinal cord injuries or other serious complications. In group B, three patients were categorized as grade A and there were two cases without neurological recovery. In the other patients, neurological function increased by 1–3 grades. In groups A and B, the postsurgical spinal stenosis rate was significantly lower than the presurgical stenosis rate (P<0.05). The postsurgical spinal stenosis rate in group B was significantly higher compared with group A (P<0.05). There was no significant difference in neurological function recovery between the groups (P>0.05). Real-time B-mode ultrasound is an effective method for posterior decompression and reduction and to observe signal changes in spinal cord blood flow in TBF.

## Introduction

With rapid industrialization, there has been an increase in the incidence of thoracolumbar burst fracture (TBF) due to traffic accidents and falls from a height, which causes an enormous economic burden on families and society. TBF often occurs in young males (20–50 years old) who engage in high-risk social activities. It accounts for ∼45% of spine fractures and is characterized by an intraspinal occupying block, which is a threat to neurological function. Spinal cord injury accounts for 30–60% of TBF cases (1).

The treatment of TBF involves relieving spinal cord compression in order to restore the diameter of the spinal canal, followed by reconstructing spinal stability. Surgery is required for the anatomical reduction of abnormalities, strong fixation and effective decompression. Surgical methods for TBF include the anterior approach, the posterior approach and the anterior-posterior combined approach. With the anterior approach, the surgery is conducted under direct vision and the intraspinal compression may be removed directly, with thorough decompression. Furthermore, intervertebral bone grafting and fusion and internal fixation may be performed to allow for the reconstruction of spinal stability and the retention of structural integrity in the posterior spine, with a high fusion rate. However, one spinal motor unit is damaged during surgery, combined with trauma and bleeding, leading to a visceral vascular injury. The posterior approach is performed when there is serious damage to the posterior spine. The posterior approach is capable of detecting the spinal cord and treating other combined intraspinal injuries with a short fixed segment. Therefore, spinal motor function may be retained to a large extent. This approach has the advantage of being a short, simple surgery with little trauma and bleeding. Posterolateral bone grafting may be performed simultaneously. The restriction in anterior decompression is the main disadvantage of this approach (2,3). Incomplete decompression (where the stenosis remains in the spinal canal), poor restoration of vertebral height and kyphosis following the removal of implanted material are common features of the anterior and posterior approaches. Early degeneration of adjacent segments and lower back pain due to bone grafting and fusion remain problematic.

Posterior short-segment fixation is a widely accepted approach for TBF. As the postsurgical vertebral bone defect may cause a lack of support in the anterior and middle spine, the failure rate of the surgery remains high. A number of improved techniques and methods for this approach have been proposed and applied in clinical research (4,5), but the efficacies require further observation.

B-mode ultrasound has the advantage of real-time imaging. Certain researchers use B-mode ultrasound and MRI to detect imaging signal changes following spinal cord injury. The difference between the spinal cord surgical injury range and the abnormal MRI signal range is comparatively studied, and the glial scar border is preliminarily positioned to determine the scope of the surgery. Furthermore, according to the real-time imaging feature of B-mode ultrasound, the glial scar is precisely positioned and completely resected. Real-time B-mode ultra-sound has been used for fracture reduction. Under the real-time assistance of B-mode ultrasound, the decompression and restoration of vertebral body height and spinal canal morphology are performed. Short-segment fixation is performed, followed by pedicle screw placement on the fractured vertebral side and vertebral pedicle autogenous bone grafting is performed on the other side. Thus, the ‘shell’ phenomenon is avoided (6–8). Furthermore, unilateral vertebral pedicle fixation and bone grafting may improve the biomechanical stability of the affected vertebral body, effectively reconstructing the support in the anterior and middle spine, reducing the stress load on the fixation system and preventing early adjacent segment regression, kyphosis and lumbar back pain.

In this study, under the assistance of real-time B-mode ultrasound, we performed posterior fenestration of vertebral lamina and bone grafting of the vertebral body via the pedicle for patients with TBF. The objective of this study was to investigate the role of real-time B-mode ultrasound in posterior decompression and reduction and to observe the signal changes in spinal cord blood flow in TBF patients.

## Materials and methods

### General data

Between February 2004 and December 2008, 138 TBF patients from The Ordos Center Hospital (Ordos, China) were enrolled in this study. They were divided into group A and group B. In group A (108 patients), there were 80 males and 28 females aged between 18–60 years, with an average age of 38 years. The patients were divided into group A and group B. In group A (108 patients) there were 80 males and 28 females. In group B (30 patients) there were 24 males and 6 females. Injury factors were as follows: traffic accidents, 45 cases; falling from height, 40 cases; and other injuries, 23 cases. The fracture segments were T12-L4. Fracture types by computed tomography (CT) were as follows: type A, 40 cases; type B, 45 cases; and type C, 23 cases. There were ten cases with traumatic shock, 18 cases with fractures in the pelvis and other limbs and 95 cases with neurological symptoms. According to the Frankel grading scale (9), 12 patients were diagnosed as grade A, 20 as grade B, 38 as grade C, 15 as grade D and 23 as grade E. In group B (30 cases), there were 22 males and 8 females aged between 20–58 years. Injury factors were as follows: traffic accidents, 10 cases; falling from height, 18 cases; and other injuries, 2 cases. The fracture segments were T12-L4. Fracture types were determined by CT; there were 12 type A cases, eight type B and ten type C. There were five cases with traumatic shock, four cases with fracture in the pelvis and other limbs and 25 cases with neurological symptoms. According to the Frankel grading scale, three patients were diagnosed as grade A, seven as grade B, ten as grade C, five as grade D and five as grade E.

Follow-up was conducted between 18 months and four years. Presurgical X-ray, CT and MRI examination revealed that there were varying degrees of traumatic spinal stenosis in all patients, with spinal cord compression in the injured spine analyzed using MRI. The criteria for intraspinal fracture piece by CT were as follows (3,4): type A, volume of fracture piece in the spinal canal accounted for <30% of the volume of the spinal canal; type B, the volume of fracture piece in the spinal canal accounted for 30–50% of the volume of the spinal canal; type C, the volume of the fracture piece in the spinal canal accounted for >50% of the volume of the spinal canal. Presurgical and postsurgical recovery of neurological function was evaluated according to American Spinal Injury Association (ASIA)standards, and the spinal decompression range was determined by measuring the proportion of the encroaching fracture piece in the spinal canal (spinal stenosis rate) on the CT image. Written informed consent was obtained from all patients.

### Surgical methods

Patients lay in a prone position and following general anesthesia, a posterior midline incision, centered by the injured vertebra, was performed to expose the injured vertebra and adjacent vertebral pedicles. We implanted five screws in one side of the injured vertebra and the adjacent vertebral pedicles, respectively. Under fluoroscopy of the C-arm X-ray machine (GE OEC Flurostar 7900; GE OEC medical system Inc., Salt Lake City, UT, USA), the insertion length and direction of the pedicle screw were adjusted. In group A, fenestration of the vertebral lamina was performed on one side, followed by observing the correlation between the fracture piece and the spinal dura mater using real-time B-mode ultrasound (10) (Aloka Alpha 10; UST-9128 probe, 5 MHz, Aloka Ltd., Co., Tokyo, Japan; or GE Vivid 7.0, 10S probe, 10 MHz; GE Healthcare Co., Nobelsville, IN, USA). The fracture piece was pushed into the vertebral body using the L-shaped operative tool until the cortical bone margin of the fracture piece was parallel to the posterior margin of the vertebral body, followed by longitudinal distraction of the vertebral body anterior margin. When a successful reduction was observed under fluoroscopy, fixation was performed. Following the pedicle bone grafting on the other pedicle of the injured vertebra column, an autologous iliac bone fragment (or allograft bone) was implanted into the hollow cavity of the injured vertebra and then fixed. The decompression status and correlation between the fracture piece and the posterior margin of the vertebral body were explored by B-mode ultrasound, until a satisfactory result was obtained.

**Figure 1.**
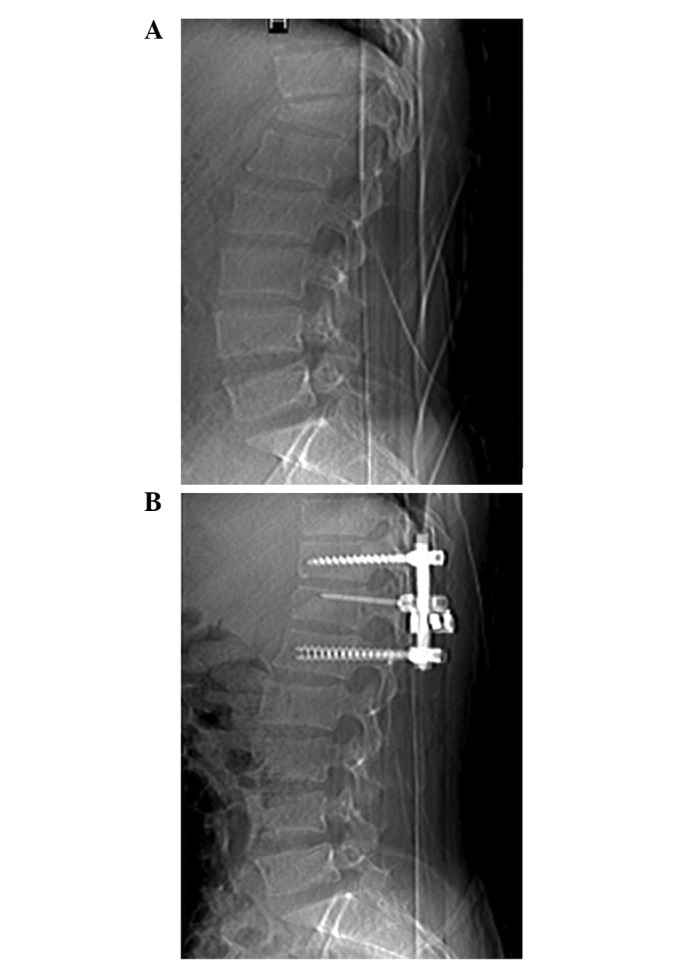
(A) X-ray normotopia and lateral projection (presurgery) from group A. (B) X-ray normotopia and lateral projection (postsurgery).

**Figure 2.**
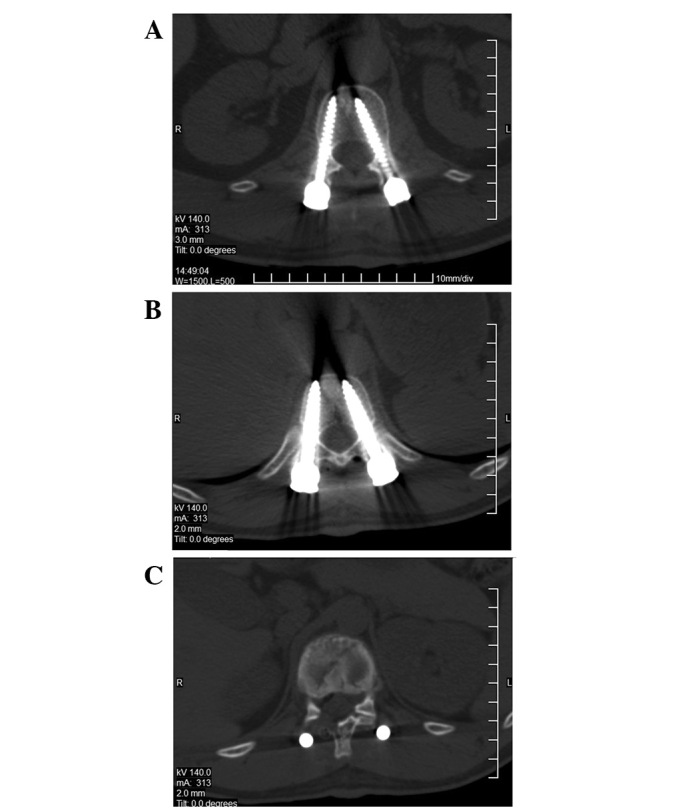
Group A two weeks after surgery. (A) CT of upper vertebral pedicle screw of injured vertebra; (B) CT of lower vertebral pedicle screw of injured vertebra; (C) CT of burst fracture vertebra (lumbar 1 burst fracture).

**Figure 3.**
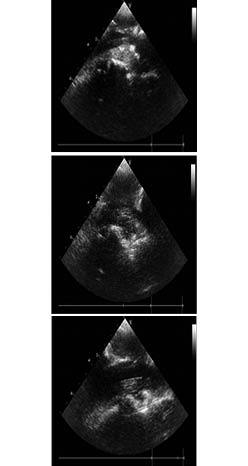
Intraoperative B-mode ultrasound.

In group B, five screws were implanted in one side of the injured vertebra and the adjacent vertebral pedicles, respectively. Bone grafting was performed on one side of the vertebral pedicle of the injured vertebra, with fenestration of the opposite vertebral lamina. An L-shaped operative tool pushed the fracture piece into the vertebral body. Once the incision was cleaned and the drainage tube had been placed, the incision was closed. Presurgical and postsurgical X-rays are shown in Fig. 1. The postsurgical CT projections after two weeks are shown in Fig. 2. The intraoperative B-mode ultrasounds are shown in Fig. 3.

### Statistical analysis

Data are expressed as the mean ± SD. Statistical analysis was performed using SPSS 11.0 statistical software (SPSS, Chicago, IL, USA). A t-test was used to analyze the differences between the groups. P<0.05 was considered to indicate a statistically significant difference.

## Results

Follow-up results revealed that, in group A, 12 patients were classified as grade A and there were six cases without neurological recovery. In the other patients, neurological function increased by 1–3 grades. There were no aggravated spinal cord injuries or other serious complications. In group B, three patients were classified as grade A and there were two cases without neurological recovery. In the other patients, neurological function increased by 1–3 grades. Table I shows that, in groups A and B, the postsurgical spinal stenosis rate was significantly lower compared with the presurgical stenosis rate (P<0.05). The postsurgical spinal stenosis rate in group B was significantly higher compared with group A (P<0.05). There was no significant difference in neurological function recovery between the groups (P>0.05; Table II).

## Discussion

Although the necessity for decompression of fracture pieces encroaching on the spinal canal in TBF patients has been a controversial topic in recent years, the majority of researchers believe that effective spinal canal decompression is important for the recovery of spinal cord function, and it is the current treatment principle for spine and spinal cord injury. It has been proposed that spinal cord injuries do not correlate positively with the state of spinal cord compression under imaging examination, but are dependent upon the degree of injury (11,12). Cases with light epidural compression but severe spinal cord injury are often observed clinically. However, evidence has suggested that the degree of spinal cord compression demonstrated by imaging correlates closely with the recovery of spinal cord function.

This study demonstrated that spinal canal decompression may be performed to treat TBF with spinal cord injury, particularly for incomplete spinal cord injuries. Therefore, it remains a method of therapy, even for TBF with clear spinal cord compression. The posterior approach may detect the spinal cord and treat other combined intraspinal injuries using a short fixed segment. It has the advantage of being a short, simple surgery with little trauma and bleeding. For patients with multiple traumas, particularly those accompanied by multiple physical injuries, the posterior approach is particularly advantageous. Without considering the recovery of neurological function, it may accelerate the treatment of other injuries. However, some fracture piece remains after the posterior approach, which interferes with the spinal cord.

There are three methods for performing fracture piece reduction or decompression in the posterior approach (13–15): i) The indirect reduction method. Traction is reduced on the fractured vertebral body using equipment without direct decompression. The fracture piece was pressed into the fractured vertebral body using the elastic tension of the posterior longitudinal ligament. This method is simple, but there is a poor level of reduction; ii) the fracture piece is not completely removed, but it is pushed into the fractured vertebral body using a bone punch. This method is relatively simple with less bleeding. However, for a big fracture piece (sagittally occupying >50%), exposing the fracture piece is difficult and may cause spinal cord injury during the separation process; iii) annular or subannular decompression, combined with separating, cutting and removal of the fracture piece. This method has a good decompression effect, and is capable of restoring the volume of the spinal canal to its maximum. In this method, the fracture piece may be separated and cut layer by layer without exposing the top of the spinal canal. It has little effect on the spinal cord and is not limited by the duration following injury (16–18). Greater intraoperative bleeding and a higher skill requirement are disadvantages of this method. Furthermore, the range of resection is greater than the normal posterior border of the fractured vertebral body, resulting in a loss of bone mass (19–21).

For TBF with serious collapse following posterior vertebral distraction and reduction, the trabecular bone and nucleus pulposus of the fractured vertebral body are not completely restored, with the existence of the ‘shell’ phenomenon (22,23). In this study, for the 108 patients in group A, real-time B-mode ultrasound assisted posterior decompression and reduction were performed and the morphological changes to spine were observed. For the 30 patients in group B, posterior fenestration combined with pushing the fracture piece into the fractured vertebral body using the L-shaped operative tool was performed. Certain researchers use B-mode ultrasound and MRI in order to detect the imaging signal changes following spinal cord injury (24). The difference between the spinal cord surgical injury range and the MRI abnormal signal range is comparatively studied, and the glial scar border is preliminarily positioned. However, according to the real-time imaging feature of B-mode ultrasound, the glial scar is precisely positioned and ultimately completely resected. In group A of this study, the real-time B-mode ultrasound detected and directed the reduction of the fracture piece and observed the pulsation and change in blood flow of the spinal cord. In group B, post-surgical CT observation revealed that the reduction effect of the fracture piece was reduced compared with group A, with a significantly higher spinal stenosis rate. During the observation period, the neurological function recoveries in the groups were not significantly different, which may be due to the small sample size.

In conclusion, neurological deficits following spinal cord injury may cause various levels of somatic dysfunction, with paraplegia in severe cases, which leads to an enormous economic burden on families and society (25,26). The selection of suitable surgical methods contributes to the rehabilitation of the patient and a reduction in postsurgical complications to the maximum extent. For TBF treatment, posterior decompression and internal fixation assisted by real-time B-mode ultrasound may shorten hospitalization time, reduce mortality, protect the spinal motor function unit, prevent early spinal degeneration and is the most suitable method for the posterior approach.

## Figures and Tables

**Table I.** Comparison of the spinal stenosis rate between the groups (%).

Group	Type	CT fracture type		
		A	B	C
A	Presurgical	18.9±3.5	39.8±4.4	56.6±4.2
	Postsurgical	2.5±2.3	1.9±2.3	1.2±3.5
B	Presurgical	19.2±3.3	38.0±4.8	55.8±3.2
	Postsurgical	6.4±1.2	5.9±1.9	8.4±4.2

P<0.05, postsurgical compared with presurgical; P<0.05, postsurgical group A compared with group B. CT, computed tomography.

**Table II.** Comparison of neurological function recovery between two groups.

Group	Type	Frankel grade				
		A	B	C	D	E
A	Presurgical	12	20	38	15	23
	Follow-up	6	10	12	20	60
B	Presurgical	3	7	10	5	5
	Follow-up	2	3	4	6	15

P<0.05, postsurgical compared with presurgical; P>0.05, postsurgical group A compared with group B.
